# Serosal membrane tuberculosis in Iran: A comprehensive review of evidences

**DOI:** 10.1016/j.jctube.2023.100354

**Published:** 2023-02-23

**Authors:** Azadeh Ebrahimzadeh, Abdol Sattar Pagheh, Tahoora Mousavi, Maryam Fathi, Sayyed Gholamreza Mortazavi Moghaddam

**Affiliations:** a Infectious Diseases Research Center, Birjand University of Medical Sciences, Birjand, Iran; b Molecular and Cell Biology Research Center (MCBRC), Hemoglobinopathy Institute, Mazandaran University of Medical Sciences, Sari, Iran; c Parasitology Department of Medical School, Tarbiat Modares University, Tehran, Iran; d Cardiovascular Diseases Research Center, Birjand University of Medical Sciences, Birjand, Iran

**Keywords:** Peritoneal tuberculosis, Pericardial tuberculosis, Pleural tuberculosis, Iran, Tuberculosis empirical therapy

## Abstract

Tuberculosis (TB) is among the most common cause of serositis. There are many uncertainties in diagnostic and therapeutic approach to serous membranes tuberculosis. Our aim in the present review is to discuss the regional facilities for timely diagnosis, rapid decision-making and appropriate treatment regarding to serous membranes tuberculosis; with emphasis on situation in Iran. A comprehensive literature searches about the status of serous membranes tuberculosis in Iran were performed in English databases including Google Scholar, Science Direct, Scopus, Pub Med, and Web of Sciences, Persian SID databases, between 2000 and 2021. The main findings of the present review are as follow: a) pleural tuberculosis is more common than pericardial or peritoneal tuberculosis. b) Clinical manifestations are non-specific and so non-diagnostic. c) Smear and culture, PCR and characteristic granulomatous reaction have been used for definitive TB diagnosis by physicians. d) With Adenosine Deaminase Assays and Interferon-Gamma Release Assays in mononuclear dominant fluid, a possible diagnosis of TB is proposed by experienced physicians in Iran. e) In area of endemic for tuberculosis including Iran, a possible diagnosis of TB is enough to begin empirical treatment. f) In patients with uncomplicated tuberculosis serositis, treatment is similar to pulmonary tuberculosis. First line drugs are prescribed unless evidence of MDR-TB is detected. g) The prevalence of drug resistant tuberculosis (MDR-TB) in Iran is between 1% and 6%, and are treated by empirical standardized treatment. h) It is not known whether adjuvant corticosteroids are effective in preventing long term complication. i) Surgery may be recommended for MDR-TB. Tamponade or constrictive pericarditis and intestinal obstruction. In conclusion, it is recommended to consider serosal tuberculosis in patients who have unknown mononuclear dominant effusion and prolonged constitutional symptoms. Experimental treatment with first line anti-TB drugs can be started based on possible diagnostic findings.

## 1 Introduction

Mycobacterium tuberculosis (M. tuberculosis) is placed in the family of Mycobacteriaceae. Humans are the only source of M. tuberculosis. It is primarily transmitted from one infected to non-infected person through inhalation of contaminated particles. The 30 countries with high tuberculosis (TB) burden account for more than 90 % of cases. Among the highest burden are India(26%), Indonesia(8.5%),China (8.4%), Philippines (6.0%), Pakistan (5.7%), Nigeria (4.4%), Bangladesh (3.6%), and South Africa (3.6%) [1]. Due to covid-19 outbreak in recent years, the report of new cases with tuberculosis was significantly declined in 2020, compared to before. Changes in supply and demand in relation to TB diagnostic and treatment are possible reasons for declining of TB report in the recent years [2].

More than 80% of TB cases are pulmonary. Serous membranes are among the common forms of extra-pulmonary tuberculosis. After lymph node tuberculosis, the most common type of extrapulmonary tuberculosis is pleural tuberculosis [3], [4]. Tuberculosis is also the most common cause of mononuclear dominant exudative pleural effusion after malignancy in Iran [5]. The prevalence of tuberculous pericarditis is relatively rare, and associated with long term morbidity and even mortality [6]. Tuberculous peritonitis is the most common forms of abdominal tuberculosis and account for 6.1% of all extra-pulmonary tuberculosis in the United States in last decade [7].

There are many conflicts in diagnosing and treating of serosal tuberculosis. Variations in outbreak reports of serious tuberculosis are affected by diagnostic facilities and also overall prevalence of tuberculosis in each region [6]. Lack of a comprehensive diagnostic approach leads to delay in diagnosis and ultimately serious complications. The purpose of the present review is to discuss the facilities for timely diagnosis, accurate decision making and prompt treatment of serous membranes tuberculosis in Iran.

## 2 Methods

A comprehensive literature searches about the status of serous membranes tuberculosis; with emphasis on situation in Iran was performed in English databases including Google Scholar, Science Direct, Scopus, Pub Med, and Web of Sciences, Persian SID databases, between 2000 and 2021. In order to access the original research as much as possible, all articles related to tuberculosis peritonitis, pericarditis, and pleuritic were reviewed. Exclusion criteria were studies conducted before 2000 and those studies on serositis unrelated to tuberculosis. The search strategy was performed using medical subject headings (MeSH) terms and the keywords: “tuberculous peritonitis”, “tuberculous pericarditis”, “constrictive pericarditis”, and “tuberculosis pleurisy” alone/or in combination with each other. First the titles of the articles, then their abstracts were reviewed. Then, the main texts of the selected articles were studied and the desired data were extracted.

### 2.1 Prevalence of serosal tuberculosis

Tuberculosis is considered as a major cause of pleural effusion, especially in developing countries. About 5% of all tuberculosis cases are presented with pleural effusions. There is already positive relationship between the overall prevalence of tuberculosis and tuberculosis pleurisy. In areas where tuberculosis is endemic, tuberculous pleurisy is accounted for significant number of pleural effusion with unknown origin [8]. Among all patients with tuberculosis, among patients with exudative effusion and among patients with extra-pulmonary tuberculosis, the prevalence of pleural tuberculosis varies from one region to another [9], [10], [11]. Table 1 showed the prevalence of pleural tuberculosis among patients studied in different area of Iran. Studies conducted in Iran show that pleural tuberculosis is the most common form of extrapulmonary tuberculosis after lymph node tuberculosis [12], [13], [14], [15]. When the causes of mono-nuclear dominant exudative pleural effusion are investigated in Iran, malignant effusion is the most, followed by tuberculosis [5], [16], [17], [18].

**Table 1:** Serosal tuberculosis prevalence in different area of Iran.

Geographic region	Studied population (Number)	Number/Prevalence	References
**Pleural tuberculosis**			
Mazandaran province (Northeast of Iran)	Extra pulmonary tuberculosis (843)	159 (18.9%)	Moosazadeh et al [19]
Golestan province (Northeast of Iran)	Extrapulmonary tuberculosis	741(30.5%)	Nikonajad et al [10]
Birjand (east of Iran)	Exudative Pleural effusion cases (327)	48 (14.7%)	Mortazavi-Moghaddam et al [5]
Babol (North of Iran)	Exudative pleural effusion (100)	33 (33%)	Heidari et al [16]
Mashhad (north east of Iran)	Exudative pleural effusion (142)	61 (42%)	Towhidi M et al [17]
Tehran (north-central of Iran)	Undiagnosed Exudative pleural effusion underwent blind biopsy (171)	52 (30%)	Solooki et al [18]

**Abdominal tuberculosis**			
Mazandaran province (Northeast of Iran)	Extra pulmonary tuberculosis (843)	49 (5.8%)	Moosazadeh et al [11]

**Pericardial tuberculosis**			
Mazandaran province (Northeast of Iran)	Extra pulmonary tuberculosis (843)	16 (1.89%)	Moosazadeh et al [11]
Tehran (north-central of Iran)	Patients with pericarditis (30 cases)	13(43%)	Falah et al [80]
Tehran (north-central of Iran)	Extra-pulmonary tuberculosis, childhood (14 cases)	2(14.2%)	Baghaie et al [27]
Zahedan (east of Iran)	Extra-pulmonary tuberculosis, childhood (45 cases)	3(6.6%)	Metanat et al [28]

The incidence of tuberculous pleural effusion is higher in HIV-positive patients than in HIV-negative patients [4]. Tuberculous pleural effusion is more common in young and middle-aged people. But some studies show a tendency to older ages [15], [19], [20], [5], [16], [17], [18], [21].

Abdominal tuberculosis is relatively common [22]. Abdominal tuberculosis involves the mesenteric lymph nodes, the gastrointestinal tract, and also peritoneum. Due to pasteurization of dairy products, Mycobacterium bovis is infrequent, and almost all cases of mycobacterial peritonitis are related to M. tuberculosis [23], [24]. Abdominal tuberculosis including peritoneal tuberculosis is placed in the third to sixth ranks in term of prevalence among cases of extra-pulmonary tuberculosis [11]. In contrast to pleural tuberculosis, peritoneal tuberculosis is more prevalent in younger but not in children. In one study conducted in Iran, 81% of patients with abdominal tuberculosis were under the age of 40. Tuberculous peritonitis is probably more common in women and sometimes associated with infertility [24].

Pericardial tuberculosis is frequently reported in endemic areas of tuberculosis. While 69.5% of pericarditis in South Africa is due to M. tuberculosis, in developed countries, it comprised up to 4%. Parallel to the increase in the incidence of HIV, the cases of tuberculous pericarditis also increase. [25]. Tuberculous pericarditis is in eighth to tenth rank of extra-pulmonary tuberculosis in term of prevalence in Iran. Unlike tuberculous peritonitis, tuberculous pericarditis is frequently diagnosed in children (Table 1) [11], [26], [27], [28].

### 2.2 Transmission and risk factors

The main way of *mycobacterium tuberculosis* spread is through droplets inhalation from patients with sputum productive (smear positive) pulmonary tuberculosis. The main transmission risk factors from the infected person include the severity and the presence of a cavity in the lung, close and duration of contact, delay in diagnosis, residences in poorly ventilated and without direct sunlight. The main risk factors in an individual to become infected and get the disease include HIV infection, diabetes mellitus, smoking, excess alcohol use, and malnutrition [29]. Most cases of extra-pulmonary tuberculosis originate from a primary focus in the lung. Tuberculosis screening test such as tuberculin test and gamma interferon assay are valuable in reducing the rate active tuberculosis [29].

Various studies have shown that the incidence extra-pulmonary tuberculosis decreases with age. One study conducted in Tehran shows that extra-pulmonary tuberculosis was more common in younger age (15 to 18 years) [30]. Considering all TB cases; pulmonary tuberculosis is more common in men and extra-pulmonary tuberculosis is more common in women. But extracted data from some studies in Iran show that meningeal, pleural, and bone tuberculosis are more common in men, and urogenital, lymph nodes, and breast tuberculosis are more common in women. Diabetic patients are more likely to develop pulmonary tuberculosis compared to extra-pulmonary tuberculosis. Although, in some studies, diabetes has been considered as a risk factor for extra-pulmonary tuberculosis. Patients with renal failure on dialysis are significantly at risk of tuberculosis (pulmonary as well as extra-pulmonary excluding meningeal and urogenital tract). Smoking exposes and also patients with underlying lung diseases are at risk of acquiring pulmonary tuberculosis more than extra-pulmonary tuberculosis. However, it is not a prominent risk factor in the events of pulmonary complications, including bronchial antraco-fibrotic change. One study points to the role of smoking as a risk factor in the development of tuberculous pleural effusion [31], [32].

Tuberculosis is more likely to occur in the cases of cirrhosis, malignancies, and in immune-compromised. HIV is known as a potentially strong risk factor for extra-pulmonary tuberculosis. Non-white ethnicities are more at risk of extra-pulmonary tuberculosis than other ethnic groups [20], [33], [34]. In a report from the United States of America, pleural tuberculosis is more common in immigrant patients [33].

In one study conducted in Karaj (north of Iran) it is claimed that vitamin D deficiency is a risk factor for both pulmonary and extra-pulmonary tuberculosis [35]. Immune deficiencies (HIV and AIDS), kidney failure, cirrhosis and malnutrition are major risk factors of peritoneal tuberculosis [36]. Along with all mentioned risk factors, genetic predisposition should be added to the list. Genetic polymorphism in the interleukin 1 and 1B gene have been shown to increase the risk of pleural tuberculosis. The role of interleukins in other pulmonary tuberculosis complications such as lung dysfunction and lung fibrosis has also been suggested [37], [38].

### 2.3 Immuno-pathogenesis

Serous membranes tuberculosis occurs by different mechanisms. In most cases, the primary focus of tuberculosis is in the lung. The involvement of the membranes may be due to inflammation caused by a delayed hypersensitive reaction or by bacterial invasion. There are several possibilities regarding the development of tuberculous peritonitis. Direct hematogenous dissemination to the peritoneum or spread to the mesenteric glands and then adjacent peritoneal contamination are important routs of peritoneal involvement. Furthermore; regarding the urogenital tuberculosis in women, tuberculosis bacillus may reach the peritoneum through the fallopian tubes. Intestinal tuberculosis and Peyer's patches contamination, and then the spread of tuberculosis bacillus to the peritoneum is another way in the development of tuberculosis peritonitis [39], [40].

The development of pericardial tuberculosis occurs via hematogenous seeding, lymphatic spreading, or, rarely directly from the adjacent infected focus of lung near the pericardium. After pericardial involvement, an immunological reaction occurs with the formation of tuberculous granuloma in the pericardium. Cytokines profile show that tuberculous pericarditis, like pleural tuberculosis, can be a delayed hypersensitivity reaction to *mycobacterium tuberculosis* antigens [40]. Acute tuberculous pericarditis may resolve without complications. Existence of conditions such as massive effusion, male gender and hypoalbuminemia exposes the patient to a greater risk of chronicity and adverse consequences [41].

More than 85% of cases with pericardial effusion in HIV-infected cohorts are caused by tuberculosis. HIV positive patients encounter with more aggressive and associated with more mortality in acute phase, but with lower risk of chronicity and constrictive pericarditis [25]. Pleural effusion occurs in the course of active tuberculosis and can be reactive or empyema. One study has been conducted on tuberculosis pleurisy in children and shown that male gender, empyema, and **monocytosis** in the peripheral blood were associated with greater risk of pleural complications, and TB empyema formation [42].

The older hypothesis in tuberculosis pleurisy states that the release of bacillus antigens into the pleural space triggers a delayed hypersensitive reaction and some cases recover without antibiotic treatment [43]. On the other hand, some authors propose that after the penetration of the tuberculosis bacillus into pleural space, first a neutrophilic reaction occurs. Then, with the activity of macrophages, some of the bacilli are destroyed. Finally, with intensification of the cellular immune response, most of the bacilli are destroyed and a Paucibacillary effusion or even sterile lymphocytic dominant effusion appeared [44].

### 2.4 Clinical presentation

Nonspecific or constitutional symptoms such as fever, sweating, anorexia, and weight loss are present both in pulmonary and extra pulmonary tuberculosis [45]. In case of tuberculous pleurisy, chest pain is often the first complaint. Acute onset presentation is usual in younger, but the presentation is insidious in elderly [6], [9]. Effusion is usually unilateral, although in some cases it may be bilateral. In a study, the incidence of bilateral tuberculosis pleurisy was reported significantly higher in cases who were HIV-positive [46]. Reports of massive effusions are relatively common [4], [5].

Tuberculous peritonitis may take three forms of pathologic change including wet-ascites, dry-plastic and fibrotic-fixed. While abdominal distention due to ascites is common feature of wet- ascites form, in fibrotic – fixed form, abdominal distention is associated with partial intestinal obstruction [24]. In the fibrotic dry-plastic form, abdominal masses may be palpable, which arouse suspicion of malignancy. In peritoneal tuberculosis, abdominal pain is a common symptom [47]. In patients with tuberculous peritonitis, clinical symptoms progress within a few weeks and usually with subacute presentation. In a study on 183 patients with tuberculous peritonitis, the mean duration from the onset time of symptoms to the time of diagnosis was 3 months (from 2 weeks to 9 months in various cases) [24].

Ascites, abdominal pain and tenderness, are frequently reported. Unlike acute bacterial peritonitis, abdominal pain in tuberculous peritonitis is mild and persistent and usually without rebound tenderness. There is a risk of delay in diagnosis due to ambiguous symptoms [22], [24]. Accordingly, peritoneal tuberculosis must be considered in any patient with constitutional symptoms in concomitant with subacute or chronic peritoneal irritation in area of endemic tuberculosis. In a study on patients with tuberculous peritonitis, the most common symptoms were abdominal pain, abdominal distension, weight loss, fever, and, 82% of patients had ascites [24].Ambiguity in clinical symptoms are more likely in elderly and also in cases with cirrhosis.

Presentation of tuberculosis pericarditis is insidious in most cases. In sometime the presentation is acute and leads to tamponade. Suspicion of the disease is the key to timely diagnosis [25], [48]. Constrictive pericarditis is a consequence of delay in diagnosis. Patients with tuberculous pericarditis go through 4 stages: The first stage (inflammation without effusion), is less likely to be detected. Chest pain and friction rub are the main symptoms at this stage. Sometimes electrocardiogram changes are seen in favor of pericarditis [48], [49]. The second stage (exudative) is the most common form of TB pericarditis diagnosed in clinic and sometimes associated with tamponade. In the third stage (absorptive), thick fluid with debris can be detected by echocardiography. There may be signs and symptoms of pericardial constriction. The last stage (constrictive) is accompanied by thickened and calcified pericardium without effusion. A patient with pericardial constriction has symptoms of heart failure without heart enlargement, and or pulmonary congestion [25]. Tuberculosis is the most common cause of pericardial constriction in developing countries [48]. There is no comprehensive study on clinical presentation of tuberculous pericarditis in Iran. The most published documents are case reports [49], [50], [51], [52]. In endemic areas of tuberculosis, any patient who presents with pericarditis should be evaluated for tuberculosis.

### 2.5 Diagnosis

Constitutional symptoms do not help diagnosis, and extra-pulmonary manifestations are included with numerous differential diagnosis. Laboratory tests such as high erythrocyte sedimentation rate, thrombocytosis, anemia, and monocytosis are Non-specific. Bacteriological diagnosis is possible only in a small number of patients. It is not possible to introduce a single specific and sensitive diagnostic method for tuberculosis serositis and combination of several methods concomitant with epidemiological information help us to diagnose the disease [53].

A. Mantoux test and QuantiFERON -TB test

Mantoux test (PPD test) is an inexpensive and non-invasive diagnostic skin test in patients suspected of tuberculosis. This test has an acceptable value, especially in areas with the low prevalence of tuberculosis. However, in endemic areas, due to the high prevalence of positive PPD test, its diagnostic value decreases. Immune deficiency suppresses the reactive response and leads to negative test [54]. With a history of BCG vaccination in infancy, children under 4 years of age usually have a positive PPD test. The prevalence of reactive PPD test in TB close contacts as well as in health workers is high (38%) [55]. In patients with tuberculous pleural effusion, the Mantoux test is negative in 30% of cases. There is no comprehensive report on situation of PPD test in patients with pericardial tuberculosis in Iran. In South Africa, 100% of cases with tuberculous pericarditis had reactive PPD test [48]. The situation is almost the same with peritoneal tuberculosis. In Turkey, 6 out of 9 patients with peritoneal tuberculosis had reactive PPD test [56], [57], [58]. One review study estimated that 54% of patients with peritoneal tuberculosis have positive Mantoux test [59]. One study conducted in Chaharmahal province (east of Iran) on 1424 healthy employees, showed that 28.5% of subjects had positive test (greater than15 mm induration) [57], [58]. The prevalence of positive PPD test(greater than10 mm) among child with tuberculosis pleural effusion was 66.6% in one study conducted in Tehran (Iran) [58]. Another study in Tehran showed that out of 14 patients with extrapulmonary tuberculosis, only 5(35%) cases had positive PPD test [27].

Unlike Mantoux test, the QuantiFERON test taken on whole blood (the registered trademark of an interferon gamma release assay (IGRA) for tuberculosis diagnosis) and is not affected by BCG vaccination. The agreement between the QuantiFERON and Mantoux test varied from one study to another. While some researchers consider the 100% agreement coefficient, the estimated agreement between the two test were 86% and 75% in studies conducted on patients who were candidate for kidney transplantation [60], [61], [62]. There was a little agreement between Mantoux and QuantiFERON test in children in Iran [62], [63]. QuantiFERON test does not show greater value than Mantoux test in diagnosis of latent tuberculosis in endemic areas of tuberculosis [61]. Mantoux test is considered by Sayyahfar et al as a valuable test in the evaluation of patients suspected of latent tuberculosis in children who candidate for kidney transplantation [64]. In the study conducted by Mansourzadeh et al, the sensitivity and specificity of Mantoux test in adults were 36% and 94% respectively for diagnosis of latent tuberculosis, and 80% and 94% respectively for diagnosis of active tuberculosis. They also stated that the sensitivity and specificity of QuantiFERON test were 100% and 98% respectively for active and 73% and 98% respectively for latent TB. They claim that QuantiFERON test is both more specific and more sensitive than Mantoux test to detect latent tuberculosis in adults [65]. In the study conducted by Talebi-Taher et al on health workers, it was stated that due to the history of BCG vaccination in infant in Iran, QuantiFERON test is more reliable for detecting both latent as well as active tuberculosis [66].

B. Imaging

Plain radiography and CT scanning are as essential part in evaluating patients with tuberculosis. Most cases of extra-pulmonary tuberculosis originate from a primary focus in the lung. High resolution CT scanning is more likely to show lung involvement [67]. Data from some studies are presented in Table 2.

**Table 2:** Frequency of x-ray (Chest HRCT and plain CXR) finding in different serosal tuberculosis.

**Type of serositis**	**Type of imaging**	**Abnormal finding** (**%**)	**References**
Tuberculosis peritonitis	CXR	Lung involvement (43%)	Demir et al [23]
Tuberculous pleurisy	CXR	Lung involvement 20%	Gopi et al [54]
Tuberculous pleurisy	HRCT	Lung involvement 85%	Gopi et al [54]
Tuberculous pleurisy	CXR	Lung involvement 17%	Diacon et al [104]
Tuberculous pericarditis	HRCT	Pericardial thickening and irregularity	Reuter et al [70]
Tuberculous pericarditis	HRCT	Pericardial thickening with effusion(Case report)	Hussein et al [105]
Tuberculous pericarditis	HRCT	Pericardial calcification: rare	Mayosi et al [106]
Tuberculous pericarditis	HRCT	Mediastinal adenopathy larger than 10 mm (100%)	Cherian et al [48]
Tuberculous pericarditis	HRCT	Adenopathy with hypo-dense center, merge with adjacent lymph nodes, an specific finding(50%)	Cherian et al [18]
tuberculous peritonitis in patients with renal failure	HRCT	Infiltration in 3 cases, Miliary pattern in one case, calcified granuloma in one case, and simultaneous pleural effusion in 10 cases.	Chau et al [77]
Tuberculosis peritonitis	CXR	Hilar adenopathy or scar of previous tuberculosis, pleural effusion	Sotoudehmanesh et al [47]

Abnormal chest radiography due to primary infectious focus of lung is reported from a minimum of 19% to a maximum of 83% in peritoneal tuberculosis [59]. Recent studies suggest that the frequency of lung involvement in patients with tuberculous pleurisy is probably higher than previously thought [65]. The combination of x-ray imaging (Preferably CT scanning) with ultra-sonography have been considered more specific in the diagnosis of tuberculous peritonitis [45]. CT scan is especially helpful in differentiating between peritoneal **carcinomatosis** and tuberculosis. Peritoneal thickening is uniform in tuberculosis vs peritoneal nodularity in carcinomatosis. Ultrasound more accurately shows the fibrotic septa that are common in peritoneal tuberculosis [59].

C. Fluid analysis

The first step in evaluation of patients with serositis is drawing out the fluid for diagnostic analysis. The extracted fluid is subdivided into exudative or **transudative** according to the light criteria [68]. In the case of peritonitis, serum-ascites albumin gradient (SAAG) is used instead of measuring the ratio of fluid to serum protein. The peritoneal fluids are classified as transudative in case with SAAG > 1.1 gr/dl and exudative in conditions with SAAG < 1.1gr/dl [68]. Cell counting and their differentiation help to distinguish between acute vs chronic processes. In acute processes, the percentage of neutrophils and in chronic processes, the percentage of mononuclear cells will be predominant. Acute bacterial infections as well as tuberculosis in the early stages are neutrophilic. In contrast viral infections as well as advanced stages of tuberculosis, are mononuclear dominant [69].

D. Biomarkers

Various biomarkers are used in diagnostic evaluation of serositis. Adenosine Deaminase (ADA) originate from lymphocytes, so in cases of mononuclear dominant effusion, its low levels help to rule out the diagnosis of tuberculosis. While most researchers consider a cut-off point of 40 IU/l for rule outing pleural tuberculosis, reports from different region of Iran are various, but generally in the range of 30–45 IU/l for diagnosis of peritoneal, pericardial and also pleural tuberculosis (Table 3).

**Table 3:** Studies conducted on ADA serum levels and Gamma interferon assay for diagnosis of serosal tuberculosis in Iran.

**Type of serositis**	**Region**	ADA levels (Cut of point or mean)	References
**Adenosine deaminase**			
Pleural tuberculosis	Birjand (south east of Iran)	30	Mortazavi-Moghaddam et al [5]
Pleural tuberculosis	Tehran(north of Iran)	46	Mohammadtaheri et al [78]
Tuberculosis pericarditis	Tehran(north of Iran)	35–37	Koochak et al [107]
Tuberculosis pericarditis	Tehran(north of Iran)	45	Rostamzad et al [108]

**Gamma interferon**			
Pleural seositis		90 IU/ml 69257 pg/l	Shahriar et al [109]
Pericardial serositis			Koochak et al [107]

Gamma interferon is a T- lymphocytes (T CD4+ lymphocytes) or NK cells cytokine. Measurement of gamma interferon in pleural, pericardial, or peritoneal fluids were used for evaluation of serosal tuberculosis, although sufficient sensitivity and specificity for a given level of this cytokine has not been reported [70], [71]. Different measurement methods and different diagnostic cut-off points according to the type of diseases (pleural, pericardial or peritoneum) have created a challenge for application of this method in practice.

One of the most questionable parameters in differentiation of tuberculous from non-tuberculosis peritoneal involvement is high levels of CA-125 tumor marker [72]. This becomes even more challenging when fibrotic masses are palpable [73].

Soluble Interleukin-2-Receptor level in exudative pleural effusion was reported to help in differentiation between tuberculosis from non-tuberculous pleural effusion. In Sharaki et al study, in 2013, the level of soluble Interleukin-2-Receptor was 9147 ± 3573U/ml in pleural tuberculosis vs 2724 ± 1326U/ml in non-tuberculosis pleural effusion (P < 0.01) [74]. Interleukin-27 and interleukin-6 are also elevated in pleural fluid of tuberculosis. The simultaneous increase in Il-27 and ADA are introduced as reliable predictors of tuberculosis pleural effusion [75].

E. Bacteriology and pathology

The gold standards for the diagnosis of tuberculosis are the identification of the tuberculosis bacillus in biological samples or granulomatous reaction with caseous necrosis in histopathological study. TB cultures media (traditional culture media or the BACTEC system) are time-consuming but gold standards for definite diagnosis of tuberculosis.

Tuberculosis pleural fluid acid fast staining and culture for M. tuberculosis are positive in 5% and 30% of cases, respectively. There are reports that 60% of smear positive cases are also culture positive for Mycobacterium tuberculosis and when the culture is repeated for two consecutive days, the probability of the positive culture increases [54], [76]. Immunocompromised patients are more likely to have positive smear and also positive culture. Due to concomitant lung involvement in some patients with tuberculous pleurisy, there is possibility of positive sputum smear or culture for M. tuberculosis. Sputum induction leads to the discovery of M. tuberculosis in 50% of cases. Bronchoscopy and bronchial washing increases the diagnostic yield.

Pathological examination has a high sensitivity for diagnosis of serous tuberculosis. Studies conducted in Iran show that the presence of granuloma in serous biopsy has been helpful in diagnosing tuberculosis [5], [24] Medical thoracoscopy has %100 specificity and sensitivity in the diagnosis of pleural tuberculosis [67]. In TB endemic areas, by collecting data of mononuclear dominant effusion, blind biopsy findings(granulomatous reaction), and high fluid adenosine deaminase level, the accuracy of the diagnosis for pleural tuberculosis approximates the surgical thoracoscopy [44], [68].

In case of peritoneal tuberculosis the diagnostic value of macroscopic appearance (Laparoscopic Violin sign and peritoneal infiltration) and pathological examination (granulomatous reaction) were 92% and 93% respectively [24], [47]. Peritoneal fluid smear and culture for M. tuberculosis have little sensitivity for the diagnosis of peritoneal tuberculosis [22], [56], [59]. However in patients with renal insufficiency, smear and culture are often conclusive [77].

Histopathology of biopsy (100%), PCR (60%), and ADA levels of pericardial fluid (80%) are the most common diagnostic methods used for the diagnosis of pericardial tuberculosis. The sensitivity of pericardial fluid culture in the diagnosis is about 53 to 75% [78]. Histopathological examination is more specific than sensitive for diagnosis of tuberculosis. Only 10 to 64% of cases with tuberculous pericarditis are diagnosed by histopathological examination [79]. In Falah et al. study; pericardial fluid smear and acid fast staining showed 23% sensitivity and pericardial biopsy 46% and 100% sensitivity and specificity respectively [80].

F. Molecular and antigen tests

Molecular tests are rapid, high specific but with low sensitivity to detect M. tuberculosis in serous spaces effusion. The specificity of the molecular tests in diagnosis of tuberculous pleurisy was reported up to 95% [81]. Zamirian M, et al. studied 23 cases with constrictive pericarditis, 5 of them were tuberculous pericarditis. The diagnosis was based on the presence of granuloma in the biopsy. PCR test was positive in 4 of them. None of the patients had positive acid fast staining. The agreement (Kappa = 0.455) between pathology and PCR was statistically significant [82]. PCR test on tissue sample is more sensitive than on fluid sample. [83]. In the study of Amini et al., sensitivity and specificity of PCR test on tissue samples for detecting of *M. tuberculosis* were 67.9% and 62.5% respectively [84]. False positives occur when the remnants of dead bacilli are obtained from tissue samples [59]. Molecular Xpert MTB/RIF Ultra (Xpert Ultra) and Xpert MTB/RIF are more sensitive methods. They are up to 63.8% sensitive and 100% specific in diagnosis of tuberculosis [85].There is no comprehensive study on the field of this subject in Iran.

### 2.6 Diagnostic approach

The possible diagnosis is usually suspected according to clinical evidence and some para-clinical findings. In various studies conducted in Iran, the definite diagnosis of serosal tuberculosis has been based on smear or culture, typical caseating granuloma in tissue sample, PCR test on fluid or tissue and in some cases the response to trial therapy for tuberculosis. Table 4 showed diagnostic criteria for serous tuberculosis in various studies in Iran.

**Table 4:** Diagnostic criteria for serous tuberculosis in studies in Iran.

**Type of serose involvement**	**Diagnostic criteria for tuberculosis**	**Reference**
Pericardial effusion	Culture and/or stain for acid fast bacilli, TB-PCR test, or typical caseating granuloma in pericardial tissue samples.	Koochak et al [107]
Undiagnosed exudative pleural effusion	Blind pleural biopsy, surgical biopsy, bronchoscopy, CT-guide biopsy, lymph node biopsy or treatment trial with anti-TB drugs	Solooki et al [18]
Exudative pleural effusion	Blind pleural biopsy, Ziehl–Neelsen staining of fluid sediment,	Mortazavi Moghaddam et al [5]
Exudative pleural effusion	AFB staining or cultures of the pleural fluid, bronchoalveolar lavage fluid, or pleural biopsy samples and also granulomas with caseous necrosis in biopsy specimens	Heidari B et al [16]
Patients with pericarditis	Adenosin deaminase activity (ADA), culture on Lowenstein-Jensen media, ZiehlNeelsen staining, biopsy of pericardial tissue and tuberculin test	Falah et al [26]
Massive pericardial effusion (case report)	Tuberculin skin test, acid-fast bacilli smear, and fluid polymerase chain reaction and also histological examination were negative, but trial therapy with an excellent response	Beidokhty et al [49]
Cases with pericarditis	Culture and/or stain for acid fast bacilli, TB-PCR test, typical caseating granuloma in pericardial tissue samples.	Koochak et al [107]
Cases with exudative pleural effusion	Histopathology, Ziehl-Neelsen staining of pleural fluid sediment, sputum and bronchial washing Response to trial anti-TB.	Mortazavi-Moghaddam et al [5]
Cases with exudative pleural effusion	Acid-fast bacilli in pleural fluid or, sputum, or necrotizing granuloma in biopsy specimens	Mohammadtaheri et al [78]
Primary pericarditis	Clinical, radiological, histopathological characteristics	Rostamzad et al [108]
Exudative pleural effusion	Culture of the pleural fluid, needle biopsy of the pleura or thoracoscopy	Shahriar et al [109]
Pleural effusion	acid-fast bacilli in staining or culture in pleural fluid or pleural biopsy, tuberculous granulomas in the pleural biopsy, pleural fluid PCR for tuberculosis, positive response to anti-TB treatment, positive sputum culture for tuberculosis	Shahraki Kourosh et al [74]
Constrictive pericarditis	Granulomatous reaction	Zamirian et al [82]
Lymphocytic exudative pleural effusion	Monteux tuberculin skin test, sputum examination, including AFB staining, and culture, pleural biopsy	Amini et al [84]
Chronic peritonitis	acid-fast bacilli in staining or culture, laparoscopic Violin sign, clinical and epidemiological findings, trial therapy for tuberculosis	Frootan et al [24]
Candidate cases for diagnostic laparoscopy	Macroscopic findings: adhesions, whitish granulations, thickening, hyperemia and retraction of the greater omentum and stalactic band, Pathology confirmation, excellent response to medical therapy.	Safarpor et al [110]
Tuberculous peritonitis	Laparoscopy or laparotomy with pathology of granuloma containing giant langhans type cells and central necrosis	Sotoudehmanesh et al [47]

### 2.7 Treatment and prognosis

**Fig. 1:**
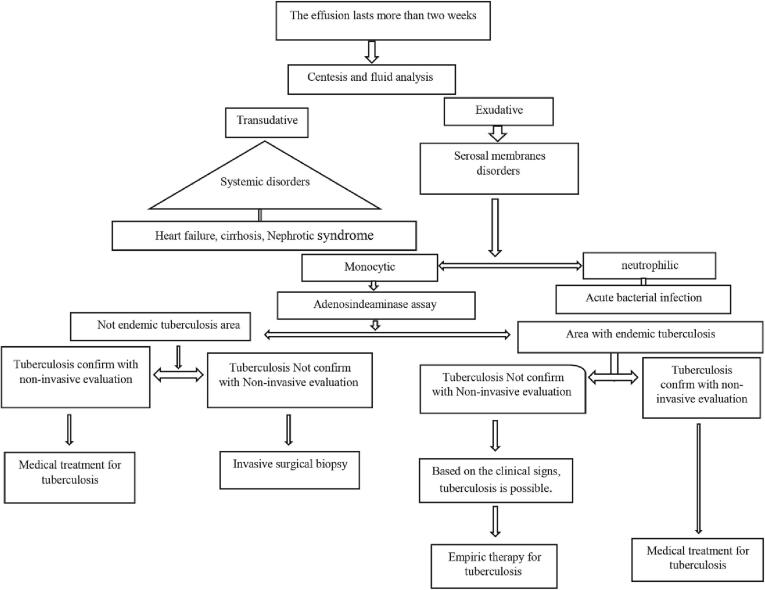
Diagnostic approaches and empirical treatment.

Therapeutic goals for serous tuberculosis are: a) Prevent the progression of the disease to other organs; b) Relieve signs and symptoms; c) Prevent the fibro destructive changes and organ dysfunction. In uncomplicated serosal tuberculosis, pharmacotherapy with anti-tuberculosis is often effective. Evidence-based diagnosis and initiation of empirical treatment are acceptable approach in endemic areas including Iran (Fig. 1). Since the prevalence of drug resistant tuberculosis in newly diagnosed cases in Iran is not high, all such cases, including serous tuberculosis, can be treated with first-line drugs [86].

The treatment protocol includes two months of the induction phase (Isoniazid, Rifampin, Ethambutol, Pyrazinamide) followed by consolidation phase for additional 4 months (Rifampin and isoniazid) [87]. The decision for starting the treatment in most studies in Iran were based on non-bacteriological findings and in some cases, response to treatment are considered as a diagnostic criterion [18]. Evacuation of pleural fluid has only a palliative role without effect on preventing complications such as fibro thorax. Intra-pleural fibrinolytic instillation in cases with complicated effusion accelerate fluid resorption and prevent adhesions [88]. Systemic corticosteroids have no effect on accelerating fluid absorption and preventing fibro thorax. Systemic corticosteroids instate are effective in damping of exaggerated systemic inflammatory reactions, which sometimes occurs within two weeks of starting treatment [89].

Treatment for peritoneal tuberculosis is essentially pharmacotherapy. Treatment is the same as for pulmonary tuberculosis. When available diagnostic procedures are limited, empirical therapy is life-saving in critically ill cases from endemic areas [24]. Studies on the beneficial effects of corticosteroids for tuberculosis peritonitis are limited. In one study, among 26 patients with tuberculosis peritonitis, daily administration of methyl prednisolone (20 mg) in 8 patients for one month in concomitant with anti-TB pharmacotherapy accelerated ascites resorption and reduced abdominal pain [23]. Frouten et al. studied 183 patients with peritoneal tuberculosis. In their study, 43 cases were treated empirically. All patients responded to the empirical treatment [24].

Tuberculosis pericarditis anti-TB pharmacotherapy is as same as pulmonary tuberculosis. It is not yet known whether adjuvant corticosteroids are effective in preventing constrictive pericarditis [90]. The European Society of Cardiology recommends adjuvant corticosteroids therapy in patients with HIV-negative pericarditis [91]. Intra-pericardial corticosteroids instillation has not preventive effect on progressing to constrictive pericarditis [6]. In cnytrast to pleural tuberculosis, pericardial drainages may be effective in preventing constrictive pericarditis [6]. In cases of constrictive pericarditis, pivotal treatment is surgery [80].

### 2.8 Prevention and control

To prevent tuberculosis spreadun, it is necessary to provide financial support for national programs and protocols in the field of health care and preventive programs. Vaccination is one of the prevention measures that are recommended for many infections. The BCG (Bacillus Calmette–Guérin (BCG) vaccine) vaccine is an attenuated M. tuberculosis bacillus with low virulence. BCG vaccination is universally implemented for newborns in Iran. The goal is to prevent miliary and meningeal tuberculosis in children. Vaccination is prohibited in cases with immune deficiency, leukemia and any cases with HIV [92].

In the preventive intervention, every effort must be made to prevent the emergence of tuberculosis with multi-drug resistant (MDR: resistant to treatment with isoniazid and rifampin), Pre Extensively drug-resistant (pre XDR –TB) and extensively drug-resistant TB (XDR-TB). About 30% of deaths due to antibiotic resistance in the world are related to drug resistant tuberculosis [93].

Improper uses of medication by the patients; incorrect dosage and incomplete course of treatment duration are the most important factors that lead to drug resistance in patients with tuberculosis. These factors provide a suitable environment for mutation. Mutated bacilli multiplied and become the dominant bacteria in the infected person's body. Mutations lead to the emergence of MDR cases [94].

Active screening, timely diagnosis, early, prompt and effective medication regimens with adherence to drug consumption and adequate treatment duration are the most important strategies for prevention of MDR and XDR. It is also very important to isolate MDR-TB cases until the risk of transmission is eliminated [95]. In the endemic areas of tuberculosis, including in Iran, diagnostic evaluations for tuberculosis are recommended in all patients who have prolonged fever with pulmonary or extra-pulmonary symptoms. History of contact with smear positive patients, especially in children, strengthens the suspicion of tuberculosis [96]. Active screening and identifying positive sputum smear patients are very important to prevent the spread of the disease. Patients who have the following conditions are considered as smear positive *mycobacterium tuberculosis* cases:

A: Two positive *mycobacterium tuberculosis* sputum smears

B: One positive sputum smear along with one positive *mycobacterium tuberculosis* sputum culture

A: One positive sputum smear along with radiographic changes in favor of tuberculosis [97].

Treatment under direct supervision (DOTs) for tuberculosis is a perfect strategy to prevent incomplete treatment and so eliminate emergence of drug resistance especially in patients who do not have acceptable compliance for taking medications, HIV positive patients, prisoners, and also patients with MDR -TB. In DOTs strategy daily medications by patients with TB are taken under the supervision of a trained health care worker or other designated individual. The success rate of treatment with DOTs is 90%, vs 70% of taking medications without direct supervision. All patients with tuberculosis in Iran are exempted from paying for treatment [98].

### 2.9 Multi and extensively drug-resistant (M/XDR-TB) and therapeutic strategies

Prevalence of primary resistance to anti-tuberculosis drugs (previously untreated person infected with MDR-TB) in the world, EMRO (Eastern Mediterranean Regional Office), and Iran are 3.4%, 4%, and 1.3% respectively. In addition, the prevalence of secondary resistance to anti-tuberculosis drugs (development of MDR-TB during treatment) in the world, EMRO, and Iran are 18%, 16%, and 8.3% respectively [99]. The prevalence of drug resistant tuberculosis in Mashhad and Tehran has been reported 4% and 6%, respectively [100]. The distribution of MDR tuberculosis is not the same in all parts of Iran. It was reported that trend of MDR-TB, particularly in re-treatment cases have been increased in last decade in Iran [101].

Although there are recommendation and emphasis on ambulatory care rather than hospitalization for MDR-TB cases, but it is conditional, and the current policy in Iran is that MDR-TB cases hospitalized in tuberculosis referral centers until the sputum smear becomes negative. Treatment strategies for drug-resistant TB cases are presented in 3 forms; (1) Standardized treatment (2) Individualized treatment: (3) Empirical (can be used for both standardized and individualized treatment). The National Tuberculosis Control Program in Iran (Except for special cases) has adopted the empirical standardized treatment strategy. In this way the treatment regimen is prescribed based on the regional prevalence of resistance to different anti-tuberculosis drugs [102].

Traditionally the following points should be considered in MDR or XDR-TB treatment.

A) There should be at least four second-line anti-TB drugs (including an injectable anti-TB drug) that are effective against MDR-TB.
B) The injectable anti-tuberculosis drug should be continued for at least 4 months after culture of the sputum becomes negative.
C) Duration of treatment extended for at least 18 months after the sputum becomes negative.

In the new 2022 guideline of the World Health Organization, new regimens in treatment of drug resistant tuberculosis have been reviewed and introduced. The most advantage of the new 2022 guideline is shortening the treatment duration. New drugs including bedaquiline (BDQ), pretomanid, linezolid and moxifloxacin (MFX) are used in these programs. Emphasizing the inclusion of BDQ in the treatment plan, the length of the treatment course is reduced to 6–9 months in cases where the extent of the disease is not severe and the patient has not been treated before. Two short-term therapeutic regimens (6 or 9 months duration) have been suggested in this regard for MDR-TB [103].

1. the 6-month BPaLM regimen (bedaquiline [BDQ], pretomanid [Pa], linezolid [LZD] and moxifloxacin [MFX]) is implemented for selected MDR-TB patients older than 15 years
2. Treatment regimens containing bedaquiline (BDQ) are preferable to treatment protocol with longer than 18 months in the following MDR -TB cases.
3. not have previously received second-line drugs
4. There is no possibility of resistance to BDQ.
5. The TB disease should not be extensive and severe.

## 3 Conclusion

To prevent irreversible complications, timely diagnosis and prompt effective anti-TB treatment must be provided for all cases with TB. Empirical treatment with first line anti-TB drugs can be started based on possible diagnostic findings in critically ill cases even without definitive diagnosis in endemic areas such as in Iran. It is recommended to consider serosal tuberculosis in patients who have mononuclear dominant pericardial or pleural effusion or ascites and prolonged fever and constitutional symptoms. Regardless of some beneficial effects of corticosteroids in treatment of tuberculous pericarditis including paradoxical inflammatory reaction following starting treatment, they have not any known effects in prevention of serosal tuberculosis complications such as constrictive pericarditis. In special condition, surgical procedures, including pericardiectomy or laparotomy to relief pericardial constriction or intestinal obstruction may be necessary. Therapeutic surgery is also may be an adjunctive to pharmacotherapy in cases with limited and not sever MDR-TB.

## 4 Author Statement

The authors confirm contribution to the paper as follows: study conception and design: Azadeh Ebrahimzadeh, and Golamreza Mortazavi Moghadam ; data collection: Abdol Sattar Pagheh; analysis and interpretation of results: Tahoora Mosuavi; draft manuscript preparation: Maryam Fathi. All authors reviewed the results and approved the final version of the manuscript.

## Declaration of Competing Interest

The authors declare that they have no known competing financial interests or personal relationships that could have appeared to influence the work reported in this paper.
